# Post-hysteroscopy Ruptured Tubo-Ovarian Abscess With Atypical Bacteremia: A Case Report

**DOI:** 10.7759/cureus.45618

**Published:** 2023-09-20

**Authors:** Jillian H Linck, Wanda I Torres, Shailja T Dayal

**Affiliations:** 1 Obstetrics and Gynecology, Nova Southeastern University Dr. Kiran C. Patel College of Osteopathic Medicine, Clearwater, USA; 2 Obstetrics and Gynecology, Suncoast Women's Care, Trinity, USA

**Keywords:** e. coli, obstetrics and gynecology, bacteremia, postoperative complications, hysteroscopy, reproductive tract infections, pelvic infections

## Abstract

Hysteroscopies are commonly performed in the diagnosis and treatment of patients with abnormal uterine bleeding. Current research suggests a low rate of all types of complications following hysteroscopies. The rate of infectious complications has been reported as exceptionally low. We present a case of tubo-ovarian abscess with *Escherichia coli* bacteremia and eventual abscess rupture in a 51-year-old gravida 3, para 2012 (G3P2) woman who underwent a hysteroscopy with concurrent polypectomy. The patient had no risk factors that have historically been attributed to the development of post-hysteroscopy infection, such as a history of pelvic inflammatory disease or endometriosis. The patient also had no known intra-operational complications that might predispose her to infection. Further, the patient’s clinical presentation was significantly atypical. Despite having *E. coli* bacteremia on admission, severe abdominal pain, lack of an adequate response to several days of intravenous broad-spectrum antibiotics, and eventually evidence of abscess rupture, the patient never met clinical criteria for sepsis, including a lack of leukocytosis. This case demonstrates a rare presentation of a rare complication and emphasizes the necessity of clinical vigilance in diagnosing and promptly treating gynecological infectious complications.

## Introduction

Hysteroscopies have been routinely utilized by gynecologists worldwide for a wide array of indications since the 1970s [1]. Common associated diagnoses include abnormal uterine bleeding (AUB) including postmenopausal bleeding, uterine polyps, and leiomyoma; common concomitant procedures include dilation and curettage (D&C), and uterine lesion destruction such as polypectomy [2]. Operative hysteroscopies are considered quite safe, with reported complication rates ranging from 1.2-3.8% for procedural failures, to less than 0.01% for infections [1]. The risk of post-hysteroscopy infection further decreases in patients with no known risk factors, such as nulliparity, active bacterial vaginosis, previous pelvic surgery, multiple sexual partners, or history of pelvic inflammatory disease [3,4]. Hysteroscopy with concomitant polypectomy has been associated with an even lower risk of complications at 0.4% as compared to concomitant endometrial resection, myomectomy, and adhesiolysis [5].

Leukocytosis is a common laboratory finding in many conditions, such as inflammation, burns, dehydration, and leukemia [6]. Perhaps one of the most common casual associations with leukocytosis is infection. Though leukocytosis is a nonspecific finding and not diagnostic of infection, it is associated with infection and is often present particularly if the presentation is bacteremic or septic. 

Here we present a patient with no known gynecologic risk factors who developed a pelvic abscess following an operative hysteroscopy with D&C and polypectomy. Further, despite rupturing of the abscess with visualization of free purulent fluid in the pelvic cavity and evidence of bacteremia upon culture, the patient did not develop leukocytosis or elevated bands at any point prior to her case culminating in an emergent exploratory laparotomy and hysterectomy with bilateral salpingo-oophorectomy. 

## Case presentation

A 51-year-old gravida 3, para 2012 (G3P2) woman developed heavy menstrual bleeding. Her gynecological medical history was largely unremarkable, including being negative for a history of abnormal pap smears, sexually transmitted infections, endometriosis, or ovarian cysts. Her obstetrical history included two vaginal deliveries, as well as one abortion. The patient was not on any daily medications.

After undergoing a pelvic ultrasound in which the ovaries were not visualized, the patient was diagnosed with AUB secondary to leiomyomas and a single polypoid structure within the endometrial cavity. 

Due to these findings, a D&C hysteroscopy and polypectomy were performed. Endometrial polyps measuring 2 cm x 2 cm x 0.3 cm and 2.2 cm x 2.2 cm x 0.4 cm were removed, the pathology for which revealed benign endometrial polyps in the background of benign endometrial mucosa. Noted, there were also prominent fragments of myometrium that could have represented a submucosal leiomyoma. The endocervical curettage removed was benign. The patient tolerated the procedure well and was discharged in stable condition on the same day as the procedure. 

**Figure 1:**
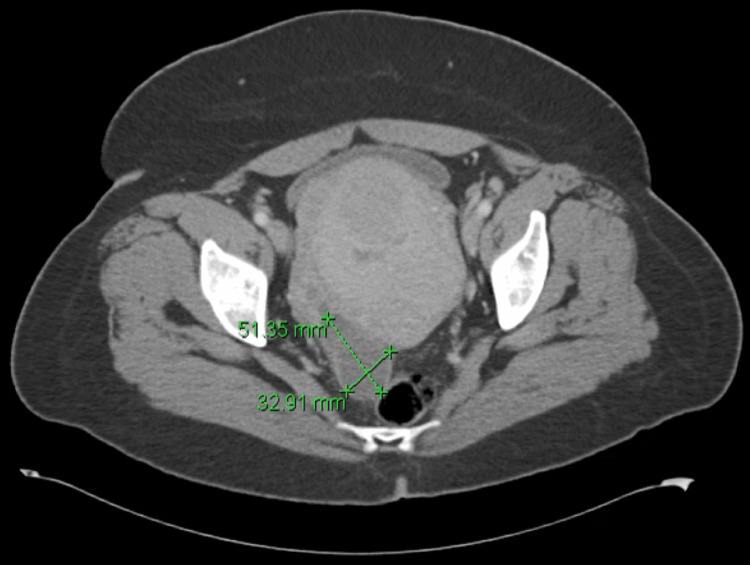
The patient's CT abdomen and pelvis at admission shows a right posterior pelvic fluid collection measuring 32.91 mm x 51.53 mm consistent with a TOA. CT: Computed tomography; TOA: Tubo-ovarian abscess

Approximately 72 hours status-post discharge, the patient presented to the hospital emergency department complaining of significant pelvic pain for the preceding 48 hours and subjective fevers. With the exception of a body temperature of 38 degrees Celsius, vitals taken in the emergency department were within normal limits including a respiratory rate of 18 breaths per minute and a heart rate of 69 beats per minute. Blood cultures and labs were drawn, and empiric broad-spectrum antibiotic therapy with piperacillin/tazobactam was initiated. The patient did not have evidence of leukocytosis, with a total white blood cell (WBC) count of 7.0 x 10^9^/L and no evidence of bandemia. No laboratory evidence of organ dysfunction was present, with a complete metabolic panel (CMP) within normal limits. Segmented neutrophils were elevated at 94%. A Computed Tomography (CT) scan of the abdomen and pelvis revealed a right posterior pelvic fluid collection measuring 3.3 cm x 5.1 cm x 3.0 cm consistent with a tubo-ovarian abscess (TOA), as well as redemonstrating an enlarged fibroid uterus (Figure 1). The patient was admitted to inpatient services. Preliminary blood culture results within 24 hours revealed bacteremia with gram-negative rods, and final results forty-eight hours later revealed *Escherichia coli* bacteremia. 

Upon initiating intravenous empiric antibiotics, the patient became afebrile within 4 hours. An inpatient interventional radiology (IR) consult was placed for assessment for drainage of the abscess noted on her CT. However, upon examination of the patient and imaging, her diagnosis was amended by IR to be a complex ovarian cyst and not indicated for drainage. The patient remained on inpatient service and the Infectious Disease team was consulted, who characterized the patient’s presentation as clinical sepsis, agreed with continuing empiric piperacillin/tazobactam, and recommended that a repeat CT pelvis be performed in a few days. 

**Figure 2:**
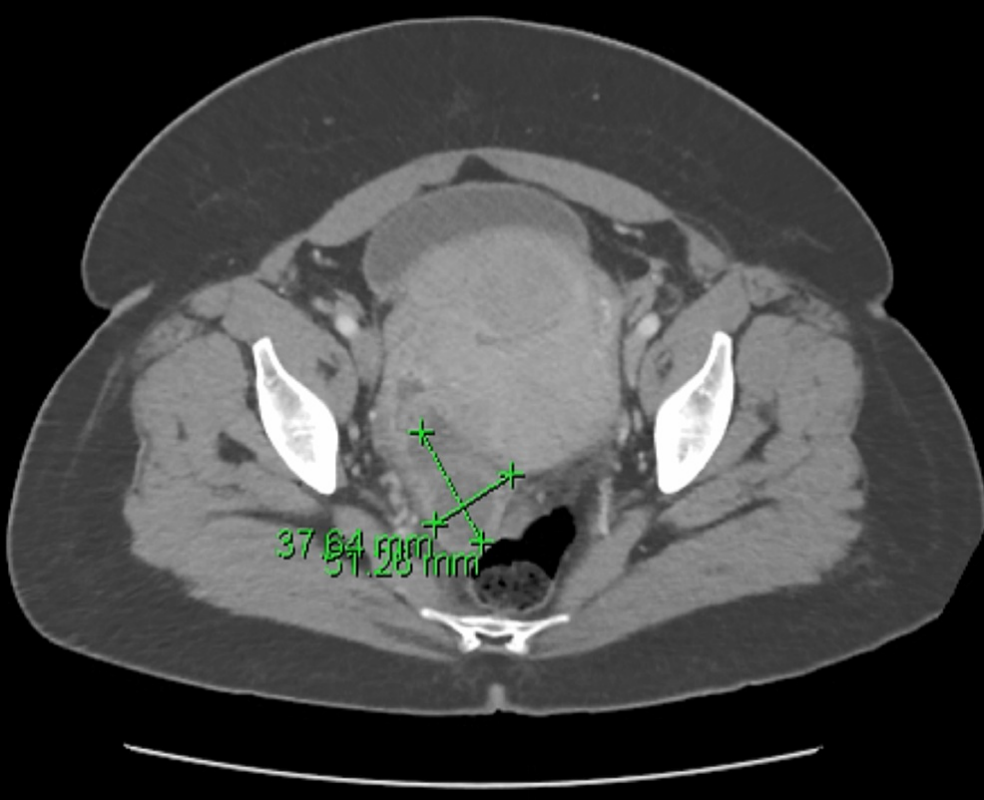
A repeat CT abdomen and pelvis shows slight interval enlargement of the fluid collection, now measuring 37.64 mm x 51.26 mm. CT: Computed tomography

On day four of hospital admission, the patient’s body temperature spiked to 38.1 degrees Celsius which was accompanied by an acute worsening of pelvic pain. Despite clinical worsening, repeat labs still demonstrated no leukocytosis, with a total WBC count only rising to 10.2 x 10^9^/L and still no evidence of bandemia or organ dysfunction within a CMP. Infectious Disease was again consulted, who changed piperacillin/tazobactam to meropenem. A stat repeat CT was ordered, which revealed a slight interval enlargement of the fluid collection posterior to and right of the uterus that may have been surrounding the right ovary (Figure 2). Additionally, a new small collection of fluid in the right paracolic gutter was present as well as a small amount of fluid in the endometrial cavity. There was no evidence of bowel injury on the image, which additionally redemonstrated multiple uterine fibroids. The patient underwent IR percutaneous drainage of the pelvic abscess on the same day with no obvious complications. 

Despite the completion of the drainage, the patient continued to suffer severe abdominal pain. Her body temperature continued to rise, measuring at 38.9 Celsius three hours status-post drainage procedure. Her leukocyte count remained within normal limits, decreasing slightly to 10.1 x 10^9^/L with still no evidence of elevated bands. At this point, it was decided that an emergent exploratory laparotomy was warranted due to acute abdomen and worsening clinical picture. Upon entry, the pelvis was filled with purulent fluid, and a total hysterectomy with bilateral salpingo-oophorectomy was performed. The post-operative pathology report revealed right fallopian tube abscess formation and transmural acute inflammation, acute serositis and transmural inflammation with a diminishing lumen of the left fallopian tube, focal acute serositis, leiomyoma and lipoleiomyoma of the uterus with benign endometrium, and a left ovary with hemorrhagic cyst and acute serositis. 

**Figure 3:**
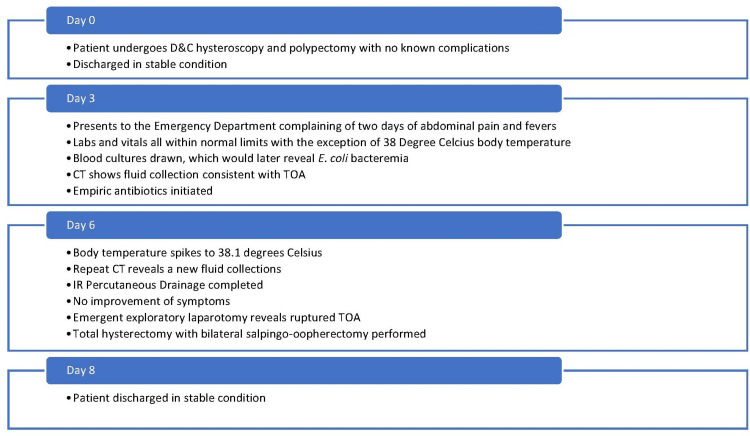
Timeline of the clinical course D&C: Dilation & curettage; CT: Computed tomography; TOA: Tubo-ovarian abscess

The patient tolerated the final procedure well and remained afebrile throughout the rest of her hospital course (Figure 3). She was discharged 72 hours later with a 14-day course of amoxicillin/clavulanate potassium. 

## Discussion

We describe a patient with no immunocompromising or comorbid risk factors who developed a pelvic abscess with eventual rupture after undergoing a D&C hysteroscopy and polypectomy. Her presentation is not only unique due to the extreme rarity of this type of significant complication, but also due to her lack of leukocytosis or elevated bands at any point during her pre-hysterectomy hospital course despite early evidence of *E. coli* bacteremia as well as later findings of free purulent fluid in the pelvic cavity. 

The risk of infection after operative hysteroscopy is reported to be under 1% based on a myriad of retrospective studies [7,8]. Prophylactic antibiotics are not routinely recommended prior to operative hysteroscopy due to the rarity of postoperative infection [7].

One reported case of TOA and subsequent sepsis with* E. coli *bacteremia following an in-office hysteroscopy was within a patient with bilateral endometriomas, which have been implicated as an independent risk factor for the development of TOAs due to the presence of stagnant blood [9,10]. Other cases include an abscess following an operative hysteroscopy in which uterine perforation occurred, and TOA following a hysteroscopy on a patient who later was determined to likely have previously undiagnosed endometriosis [3,11]. 

The latter three reports of patients with post hysteroscopic pelvic abscesses all demonstrated significant leukocytosis either on initial labs drawn in emergency departments or on labs drawn immediately after admission to inpatient care. In this context, it is unique that our case lacked this common finding, especially considering that the patient had no history of immunocompromising conditions or conditions that may have altered her vitals or labs in this case. In addition, the patient's pre-procedure complete blood count (CBC) values were within normal limits. Though the patient’s WBC count did eventually become elevated to 12.6 x 10^9^/L, it was not until one day after her emergent exploratory laparotomy and hysterectomy with bilateral salpingo-oophorectomy, likely representing an inflammatory response in healing from the surgery itself rather than a reaction to the bacterial infection. One frequently cited study of patients with proven bacteremia presenting at a single tertiary care center over the course of one year suggests that bacteremia frequently does not result in leukocytosis [12]. Further research on a larger scale is needed to establish the frequency of leukocytosis with bacteremia associated with TOA, especially with rupture. 

The most updated definition of sepsis is a life-threatening organ dysfunction caused by a dysregulated host response to infection [13]. Our patient was also atypical in the fact that she met neither the Systemic Inflammatory Response Syndrome (SIRS) based criteria for sepsis, nor the Quick Sequential Organ Failure Assessment (qSOFA) criteria for sepsis, and yet had a proven infection with bacteremia and presented with severe pain and a deteriorating clinical picture. The patient's heart rate never rose above 90 beats per minute, her respiratory rate never rose above 20 breaths per minute, she did not have any evidence of bandemia at any point, and she never developed leukocytosis prior to the final surgery. She also had metabolic values within normal limits, including blood urea nitrogen, creatinine, and bilirubin. Her urine output remained appropriate and she never demonstrated any signs of an altered mental status. Her only abnormal lab value was an initially elevated segmented neutrophil count, a nonspecific finding indicative of immune system activation, infection, and/or inflammation. Previously published case reports of TOAs with rupture very frequently involve classically defined sepsis according to SIRS or SOFA criteria [9,14-16]. This is especially significant in the context of the most recent published guidelines on sepsis identification and treatment, which acknowledge the variation in diagnostic accuracy of sepsis screening tools such as qSOFA and, further, excludes the establishment of SIRS criteria to define sepsis [17]. Of note, blood lactate was not drawn. This value, if elevated, can serve as a lab biomarker for sepsis, and its use is recommended as part of the hour-1 sepsis bundle for patients with established sepsis. However, the most recent sepsis guidelines specify that lactate alone lacks enough sensitivity as well as specificity to rule-in or rule-out the diagnosis of sepsis on its own [17]. Thus, in this patient, who had no other lab biomarkers of sepsis, an elevated lactate level could have certainly been a useful, yet nondiagnostic, piece of information.

Our patient did not meet the criteria for any single screening tool, and yet was treated with a level of urgency appropriate for a septic patient which was likely a factor in her generally favorable clinical outcome. This case therefore demonstrates the limitations of screening tools, a topic which is underscored in the most recent changes to the surviving sepsis guidelines.

## Conclusions

This case demonstrates that although exceedingly rare, serious infectious complications can occur after an operative hysteroscopy in a patient with no known risk factors. Early recognition of infection following operative hysteroscopy and prompt treatment may prevent worsening sequela. In this case, despite not meeting the older SIRS or newer SOFA criteria for clinical sepsis, our patient was treated in the same manner and with the same level of urgency as a classically defined septic case. This case serves as a reminder to be aware of rare complications following uncomplicated gynecologic procedures so that early treatment may be initiated. Additionally, this case demonstrates the critical importance of treating atypical bacteremic patients with proven infection in the same manner as classically defined septic patients. 
